# DIC-Enhanced Identification of Bodner–Partom Model Parameters for Bitumen Binder

**DOI:** 10.3390/ma16051856

**Published:** 2023-02-24

**Authors:** Marek Klimczak, Marcin Tekieli, Piotr Zieliński, Mateusz Strzępek

**Affiliations:** Faculty of Civil Engineering, Cracow University of Technology, Warszawska 24 Street, 31-155 Cracow, Poland

**Keywords:** digital image correlation, bitumen binder, numerical modeling

## Abstract

Bitumen binder is a component of asphalt mixtures that are commonly used as the materials constituting the upper layers of a pavement’s structure. Its main role is to cover all the remaining constituents (aggregate, filler and other possible additives) and create a stable matrix, in which they are embedded due to the adhesion forces. The long-term performance of bitumen binder is crucial to the holistic behavior of the layer made of the asphalt mixture. In this study, we use the respective methodology to identify the parameters of the well-established Bodner–Partom material model. For the purposes of its parameters identification, we carry out a number of the uniaxial tensile tests with different strain rates. The whole process is enhanced with a digital image correlation (DIC) to capture the material response in a reliable way and to provide deeper insight into the experiment results. The obtained model parameters were used to compute numerically the material response using the Bodner–Partom model. Good agreement between the experimental and numerical results was observed. The maximum error for the elongation rates equal to 6 mm/min and 50 mm/min is of order of 10%. The novel aspects of this paper are as follows: the application of the Bodner–Partom model to the bitumen binder analysis and the DIC-enhancement of the laboratory experiment.

## 1. Introduction

Bitumen binder is one of the main constituents of asphalt mixture that is a common type of material used in civil engineering. In addition to bitumen binder, the asphalt mixture is made of aggregate, filler and other possible additives. In this study, we focus solely on the performance of bitumen binder. This is due to the fact that its behavior determines in the most significant manner the overall long-term response of the asphalt mixture. In the context of the numerical modeling, the approximation of the aggregate behavior as the elastic seems to be sufficient [1,2,3,4]. On contrary, the response of the bitumen binder can be regarded as elastic only in a very limited load range. Regardless of the aging, temperature, moisture and other phenomena influencing the performance of bitumen binder, its mechanical response exhibits both elastic and inelastic character in a typical range of applications.

Many researchers have contributed to the study of bitumen binder mechanical performance. We refer to excellent review books [5,6], presenting the current state-of-the-art in the asphalt modeling in a detailed manner. There is also a considerable number of the scientific papers, which also provide a comprehensive description of the latest achievements in this active research field [7,8,9]. Despite the fact that bitumen binder is a well-known material, emerging new technologies of its production influence its performance [10,11]. Consequently, the material models used for the description of its mechanical response also need to be validated and updated when necessary.

**Figure 1:**
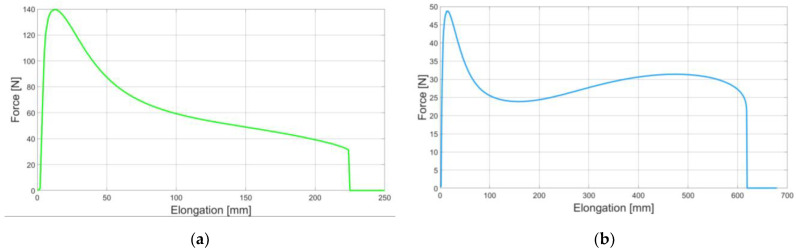
Exemplary ductilometer test results: (**a**) for neat bitumen binder; (**b**) for SBS-modified bitumen binder (own research results).

This can be illustrated with the example of neat bitumen binder and its modified counterpart. In Figure 1, one can observe the exemplary elongation–force plots obtained in a ductilometer test. On the left (Figure 1a), the results obtained for a neat bitumen binder are presented. After the maximum force was measured, the softening phenomenon was clearly visible. On the right (Figure 1b), the exemplary results obtained for a styrene-butadiene–styrene (SBS)-modified binder are shown. The plot character is different in this case. Due to the presence of the SBS modifier, after the first peak and the corresponding softening phase, a hardening phenomenon can be also observed. Both of the tests were finally performed up to rupture. It should be noted that the mechanical response to the applied load (elongation with a constant strain rate) is clearly different for these two binder types.

In the context of the numerical modeling, different constitutive equations should be adopted in order to adequately simulate the behavior of these materials. This example shows the complexity of the tackled problem in its general overview. As remarked in another study [5], there is no one general model capable of capturing all the phenomena occurring in the bitumen binder. One has to restrict the range of analysis developing even a very complex material model.

In this study, we focus on a small displacement range of the bitumen binder performance. This is justified by the mechanistic approach [12,13] to asphalt pavement structure design, in which the observed displacement quantities under a single-tire load are relatively small. Thus, the numerical analysis in the small displacement range seems to be sufficient in this case.

The main constitutive models used for the numerical modeling of bitumen binder at the continuum level in the small displacement range are far beyond the elasticity. Elastic computations are used only as the initial tests or as the part of the large multiscale framework [14]. When the research focus is on the possibly wide range of the bitumen binder performance, the assumption on its elastic response is naturally insufficient.

As reported in a previous study [15], the nonlinear mechanical asphalt behavior is influenced by the temperature, microvoid evolution as well as the rate-dependent viscoplastic hardening. In this paper, we do not investigate all of the consequences of the aforementioned phenomena. This is due to the fact that only relatively short tests (up to 1 min) at low strain levels were modeled.

In such a restricted setting, two main groups of models can be roughly distinguished:

- Viscoelastic models, which account for the recoverable asphalt deformations;
- Viscoplastic models, which account for the unrecoverable asphalt deformations.

A viscous character of the asphalt response can be easily demonstrated. For instance, in a ductilometer tests, the increase of the strain rate undisputedly results in the increase of the force value measured.

The assumption on the recovery mode of the deformation is a difference between viscoelastic and viscoplastic models. Schapery’s nonlinear viscoelasticity model [16], originating from polymer analysis, is one of the well-established models also used for the purpose of bitumen binder modeling. Linear viscoelastic Burgers or generalized Maxwell models are also frequently used for typical binder types [17]. Their advantage is a clear mechanical interpretation of the basic versions and a relatively simple numerical implementation, consequently. In the case of their versions consisting of more than one basic element, the physical interpretation of model parameters becomes infeasible. Thus, these parameters are identified using the curve-fitting.

Pure viscoplastic behavior of bitumen binder is typically modeled using Perzyna’s theory [18]. It is a classical strain rate-dependent plasticity model proposed in the 1960′s, which is based on the viscoplastic flow rule.

In many latest papers (see e.g., [15,19]), the extensions of Schapery’s [16] and Perzyna’s [18] theories are developed. This is mainly due to the new microstructural observations. For instance, in another study [15], the viscoplastic Perzyna-type model accounted for the observation that only the undamaged subdomain carries the load. In this paper, viscoelasticity and viscoplasticity were additionally coupled with a viscodamage phenomenon.

Apart from these two major groups (and their combinations) of constitutive models used in the context of the bitumen binder modeling, one can also distinguish a group of so-called unified material models [20,21], in which the overall inelastic response is modeled together, introducing the internal variable concept. These models originate from metallurgy [20,21] and were tested on a variety of metal alloys. However, they are able to simulate the typical phenomena observed in the rheology, i.e., creep and relaxation. As such, they were also applied to other kinds of materials, including technical fabrics [22] and rubber-toughened plastics [23]. Two representatives of this group of constitutive models are the Chaboche [21] and Bodner–Partom models [20]. The applicability of the latter to the numerical modeling of bitumen binder is studied in this paper. A more elaborate discussion on the reasons for this model selection is provided in the forthcoming chapter. Basically, there are three main reasons:

- It is feasible for nonlinear asphalt behavior modeling;
- Its numerical implementation is very effective due to the explicit time-integration scheme; it is particularly profitable in the case of the transient analysis—no Newton-Raphson iterations are necessary in the time-stepping algorithm.
- Its material parameters can be identified in a physically based manner, whereas typically, only a curve-fitting is used.

The aforementioned advantages of the model make it potentially robust and trustworthy in the case of asphalt numerical modeling. Moreover, the Bodner–Partom model exhibits its superiority to other popular approaches (e.g., the Burgers or Maxwell models) due to its physically based parameters and the correspondingly reliable identification procedure. In the case of the typically used models (e.g., Burgers or Maxwell), only their basic versions have a clear mechanical interpretation. Their generalized versions are the series of the number of such basic elements. The identification of such generalized model parameters is based on the curve-fitting procedure. The number of basic elements and their parameters are not determined on the basis of physical observations but are assessed in the least squares sense. Consequently, one can obtain an excellent fit of such models, but the reproducibility of the results is very limited. These parameters can be used to model only a specific specimen, for which they were identified.

The purpose of our study is to present the procedure of Bodner–Partom model parameter identification for bitumen binder. To our best knowledge, this has never been conducted before for this material solely. A similar study devoted to the whole asphalt mixture response modeled with the Bodner–Partom model can be found [24]. We point out the main difference between our study and this paper in Section 2.3.

Additionally, we present the proposed DIC enhancement of the whole methodology. It provides a more detailed insight into the analyzed material. In addition to the correction of some experiment results, the DIC can be also used to obtain the information outreaching the possibilities of the traditional test machines.

This paper is organized as follows. Section 2.1 describes samples preparation. Section 2.2 provides a brief description of the Bodner–Partom model. Section 2.3 presents the methodology for model parameter identification. In Section 3, results for the selected bitumen binder are presented and the results of the experiments and finite element calculations are compared. Discussion on the results is provided in Section 4 and concise conclusions are presented in Section 5.

## 2. Materials and Methods

### 2.1. Samples Preparation

In order to identify Bodner–Partom model parameters, the results of several uniaxial tensile tests are necessary. For the purposes of this study, such tests were performed using a ductilometer VIATECO located in the laboratory of the Chair of Highway, Railway and Traffic Engineering of Civil Engineering Faculty (Cracow University of Technology). The specimens were made of neat bitumen binder 35/50 (see Table 1 for the parameters specified by the manufacturer) and prepared for the test according to PN-EN 13398:2017-12 [25]. Subsequently, two types of experiment were performed. The first type was a shortened (continued only within the small strains range) version of the standard test performed according to [25]. Namely, the specimens were kept with a full immersion in a water at a constant temperature of 10 °C. The presence of water disabled the DIC application in this case. This was due to serious image quality deterioration because of water motion, reflexes and other undesired aspects. Thus, the second experiment type was the uniaxial tensile test without the presence of water and performed at a room temperature of 19.5 °C.

**Figure 2:**
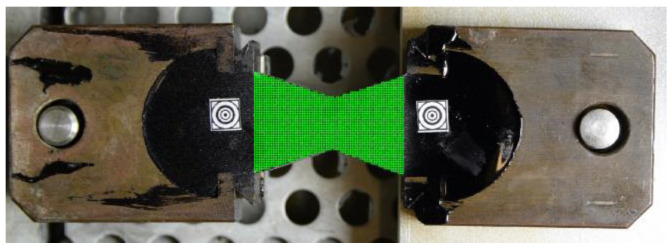
A specimen prepared for the DIC-enhanced test.

In Figure 2, one can observe a specimen with two additional markers stuck on and a virtual mesh (in green) generated in the image for the DIC purposes.

Due to the difference in temperature, the results of these two experiment types cannot be fully quantitatively compared to each other. For the sole purpose of material parameter identification, a standard experiment type is sufficient. We demonstrate a larger potential of the DIC-enhanced test, however.

The specimens used for this test need special treatment. In order to increase the image contrast, the specimens were sprayed with a black matt paint to create a very thin (but almost full) film. After drying out, a white paint was sprayed on to create a pattern for DIC purposes.

Both experiment types were performed for 4 elongation rates: 6 mm/min, 50 mm/min, 120 mm/min and 240 mm/min. Three specimens were used for every test in order to reduce the measurement error possibility. The results obtained for every triple were averaged for further processing.

### 2.2. Digital Image Correlation

Optical measurements were carried out using a digital single-lens camera (DSLR) Nikon D5300 equipped with a high-quality lens Sigma 17–50 mm f/2.8 EX DC OS HSM with negligible radial distortion in order to ensure high-quality photos, which are the basis for further processing. Photos of the sample’s surface were taken at constant intervals using an intervalometer. Each photo was 6000 pixels horizontally and 4000 pixels vertically. The camera was mounted on a tripod equipped with a micrometer head and set perpendicularly with the lens axis to the surface of the tested sample. The photos were taken with a focal length of about 24 mm with an ISO-100 value and the shutter speed was set in the range from 1/50 to 1/25 s. To ensure the ability to use such a high shutter speed and to avoid discoloration related to the color temperature strong LED light source with a temperature range of 4000–5700 K was used.

In order to ensure the possibility of determining displacements of structures in the form of subsets of a digital image of the sample surface, the digital image correlation method (DIC) was used. This method was implemented in the proprietary CivEng Vision 1.0 software developed at CUT (Cracow, Poland) by one of the paper’s author [26,27] for optical measurements and was used to process all of the photos taken. In general, the idea of using cross-correlation to measure shifts in datasets is to compare fragments of the digital image (photo) with its surroundings to determine the displacement of the examined fragment in the Cartesian coordinate system. The displacement in pixels can then be easily converted to real values in the metric system by analyzing the parameters of the lens, its distance from the sample surface or by rescaling the known size of the object included in the calibration image. By measuring the displacement of two independent subsets of the image in this way, the values of the engineering strains can be determined on a given basis. By covering the surface of the sample in the image with a grid of markers (subsets), it is also possible to determine the map (field) of displacements and, in the next step, the field of deformations. For markers tracking in this work, zero mean-normalized cross-correlation (ZNCC) was used. To ensure adequate measurement resolution at the level of traditional sensors (strain gauges), a subpixel measurement was used, obtained by interpolating pixel values to intermediate values assigned to subpixels.

### 2.3. Bodner–Partom Model

The Bodner–Partom model [20] was introduced in the 1970′s for purposes of metal alloy modeling. Its initial version was based on thermodynamic and microstructure behavior observations. The model has been actively developed since its introduction and one can find a variety of its modifications. In this paper, we briefly recall its basic version (c.f. [22,23]).

As was remarked, the model originates from metal alloy analysis. However, there is no assumption on the internal structure of the analyzed material. There are no parameters corresponding to the internal structure character. It was successfully used for the materials that exhibit in the range of small strains the same phenomena as metal alloys (e.g., polymers) but have a different internal structure. Due to the general character of the Bodner–Partom model, there were no additional assumptions necessary in the analysis of the bitumen binder. Regardless of the internal microstructure, the macroscale phenomena were modeled after the identification of the specific model parameters for the bitumen binder. Having restricted the analysis to the small strains range, we were able to model its behavior in a reliable way compared to the laboratory experiments. Identified parameters are obviously different than those present in the literature for the metal alloys, but they allowed for the reproducibility of the experiments using finite element analysis without any numerical instabilities.

The restriction to the small strain range of analysis is justified using the mechanistic approach to the design of pavement structures. Therein, small strains are observed.

In the literature, one can find the attempt of the Bodner–Partom model’s application to the modeling of cement-emulsified asphalt mixture [24]. There are two main differences between the approach developed in this study and that paper.

Firstly, we solely analyze the behavior of the bitumen binder. This is motivated by the multiscale modeling of the asphalt mixture, where all the constituents are analyzed using dedicated material models. The overall response, calculated at the macroscale, is the effective one. The results of our study can be reproduced for a variety of asphalt mixtures (different gradation scales, aggregate morphologies, etc.), since the bitumen binder parameters for the Bodner–Partom model were identified. Another approach was used in [24], where the effective model parameters were obtained for the asphalt mixture. Any modification to the asphalt mixture would require a new parameter identification to be valid for potential numerical modeling purposes.

Secondly, the identification procedure used in that study [24] is based on nonlinear curve fitting. In our study, we follow the methodology presented in other studies (e.g., [22,23]). It is a physically based approach, with many successful applications in the literature [20,22,23].

The Bodner–Partom model belongs to the group of the so-called unified models, i.e., the overall inelastic deformation (rate-dependent and rate-independent part) is modeled together, without decomposition into respective components. Additionally, there is no assumption on the existence of the yield surface in the model. Contrarily, it is assumed that the elastic deformations are accompanied with the inelastic ones regardless of the observed stress level. The lack of yield condition simplifies the numerical modeling process. The inelastic reconstruction of the material during the modeled process is characterized by the internal variable approach. One can observe an increasing number of material parameters when additional phenomena are accounted for in the model. Herein, we present a basic form of the Bodner–Partom constitutive equations that is applicable to the short-term process at the constant temperature.

The total strain rate $\dot{\varepsilon }$ is decomposed in an additive manner into the elastic ${\dot{\varepsilon }}_{e}$ and the inelastic ${\dot{\varepsilon }}_{vp}$ strain rate terms:

(1) $$\dot{\varepsilon }={\dot{\varepsilon }}_{e}+{\dot{\varepsilon }}_{vp}.$$

In the case of the uniaxial test, the inelastic strain rate term is expressed in the Bodner–Partom model as:

(2) $${\dot{\varepsilon }}_{vp}=\frac{2}{\sqrt{3}}D_{0}sgn\left(\sigma \right)exp\left[-\frac{1}{2}{\left(\frac{R+D}{\sigma }\right)}^{2n}\frac{n+1}{n}\right],$$

where $D_{0}$ is the limiting strain rate (1/s), σ is the stress (Pa), R and D denote the isotropic and kinematic hardening functions, respectively. Dimensionless constant n is the strain rate sensitivity parameter. Both isotropic and kinematic hardening functions are defined by the initial value problems (see [22,23] for more details). For the sake of brevity, we present their integrated forms herein, i.e.,

(3) $$R=R_{1}\left[1-exp\left(-m_{1}W^{I}\right)\right]+R_{0}exp\left(-m_{1}W^{I}\right),\;X=\sqrt{\frac{3}{2}}D_{1}sgn\left(\sigma \right)\left[1-exp\left(-m_{2}W^{I}\right)\right],\;D=\sqrt{\frac{2}{3}}Xsgn\left(\sigma \right),\;W^{I}=\sigma \varepsilon_{vp}.$$

In (3), X can be understood as the auxiliary variable, $R_{1}$ stands for the limiting value of the isotropic hardening (Pa), $R_{0}$ stands for the initial value of the isotropic hardening (Pa), $D_{1}$ is the limiting value of the kinematic hardening, $m_{1}$ and $m_{2}$ are the coefficients for isotropic and kinematic hardening (1/Pa), respectively. $W^{I}$ is the inelastic work.

For the sake of the model parameters identification, a functional $f_{1}$, which links the stress and the inelastic strain rate, is defined:

(4) $$f_{1}\left({\dot{\varepsilon }}_{vp}\right)=\frac{\sigma }{R+D}\;$$

In the case of the Bodner–Partom model, it has the following form:

(5) $$f_{1}={\left[\frac{2n}{n+1}ln\left(\frac{2D_{0}}{\sqrt{3}{\dot{\varepsilon }}_{vp}}\right)\right]}^{-\frac{1}{2n}}.$$

In the presented version of the Bodner–Partom model, one has to specify 7 parameters for the uniaxial tests: $D_{0}$, $D_{1}$, $R_{0}$, $R_{1}$, $m_{1}$, n, n. In addition, the Young’s modulus E (Pa) needs to be specified in order to model the elastic deformation.

### 2.4. Identification of Bodner–Partom Model Parameters

As mentioned in Section 2.1, identification of model parameters is based on the series of the experimental results and their further processing. For the sake of clarity, only the selected or averaged results are typically presented in the manuscript during the methodology explanation.

**Figure 3:**
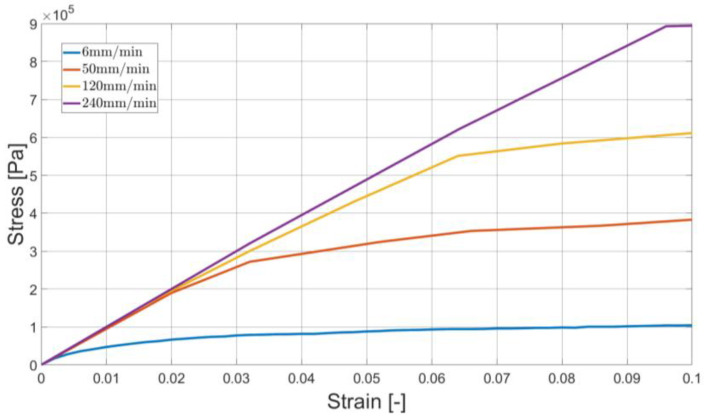
Stress–strain curves for different elongation rates (standard experiment type).

In Figure 3, one can observe a typical stress–strain relationship for various elongation rates. Herein, the results of the standard experiment are presented.

Taking into account the initial (linear) relationship, we compute the Young’s modulus (E=10,000,000 Pa). Then, the inelastic strains are computed subtracting from the total strain ε its elastic part:

(6) $$\varepsilon_{vp}=\varepsilon -\frac{\sigma }{E}\;$$

**Figure 4:**
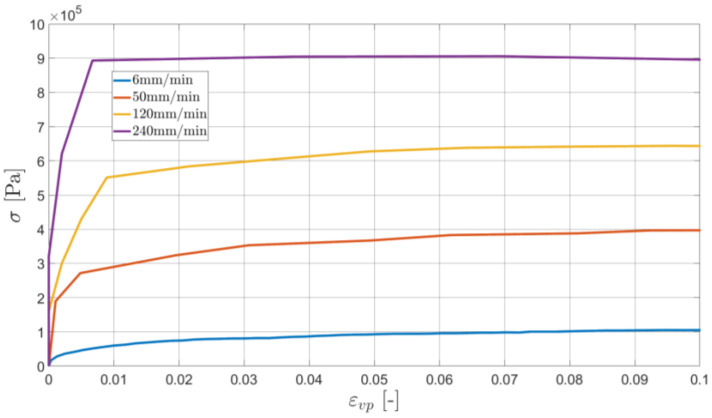
The relationship between the stress and the inelastic strain for different elongation rates (standard experiment type).

The results are shown in Figure 4.

**Figure 5:**
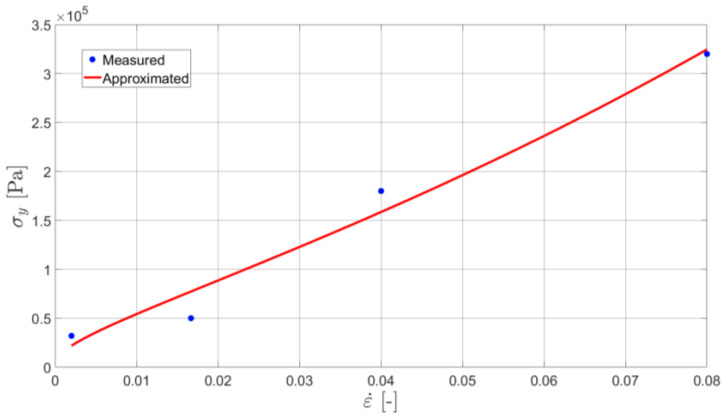
The relationship between the initial yield stress and the strain rate (standard experiment type).

An initial yield stress (being also the technical elasticity limit) $\sigma_{y02}$ is specified on the basis of Figure 4. Namely, the stress occurring at the moment of the inelastic strain equal to 0.2% is found. Using these values, which are specified for every elongation rate, one defines the relationship between the initial yield stress and the strain rate (see Figure 5).

Assuming that for the value of $\varepsilon_{vp}=0.2\%$, which was specified for the $\sigma_{y02}$ assessment, the isotropic hardening function R is approximately equal to its initial value $R_{0}$ and the kinematic hardening is negligible, one can define a closed-form expression for the technical elasticity limit rearranging Equations (4) and (5) for ${\dot{\varepsilon }}_{vp}=\dot{\varepsilon }$ at the initial level of inelastic strains ($\varepsilon_{vp}=0.2\%$):

(7) $$\sigma_{y02}={\left[\frac{2n}{n+1}ln\left(\frac{2D_{0}}{\sqrt{3}{\dot{\varepsilon }}_{02}}\right)\right]}^{-\frac{1}{2n}}R_{0}\;$$

In Equation (7), ${\dot{\varepsilon }}_{02}$ denotes the value of the total strain rate at the moment when $\varepsilon_{vp}=0.2\%$. Since the strain rates are very low, the value of the limiting strain rate $D_{0}$ is assumed to be equal to 1 s^−1^. Under this assumption, one can find the values of the parameters n and $R_{0}$. As suggested in previous studies [22,23], the Marquardt–Levensberg algorithm [28] was used to fit their values in the least-squares sense. The approximation of $\sigma_{y}$ for the specific data set is shown in Figure 4.

In order to identify $m_{1}$ and $m_{2}$ parameters, one has to define a relationship between the yield stress $\sigma \left(\varepsilon_{vp}\right)$ and the hardening work rate function γ, which is defined as:

(8) $$\gamma =\frac{d\sigma }{dW^{I}}=\frac{d\sigma }{d\varepsilon_{vp}}\frac{1}{\sigma }\;$$

Rearranging Equations (3)_III_ and (4), one obtains

(9) $$\gamma =f_{1}\left[m_{1}\left(R_{1}-R\right)+m_{2}\left(D_{1}-D\right)\right]\;$$

**Figure 6:**
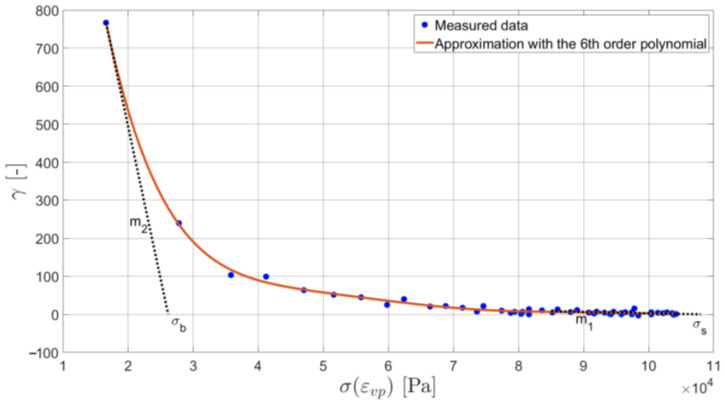
The relationship between the hardening work rate function and the yield stress.

Such a relationship, γ-$\sigma \left(\varepsilon_{vp}\right)$, needs to be created for every elongation rate and the obtained values of $m_{1}$ and $m_{2}$ parameters have to be averaged. The results plotted for the elongation rate equal to 6 mm/min in the standard experiment are presented in Figure 6.

Parameters $m_{2}$ and $m_{1}$ are the slopes of the tangents to the hardening work rate function corresponding to the inelastic strain initiation ($m_{2}$) and to its saturation ($m_{1}$). Yield stress values at the intersection points of these tangents with the horizontal axis are $\sigma_{b}$ and $\sigma_{s}$ (see Figure 5). They are used for the assessment of the remaining two parameters of the Bodner–Partom model.

Finally, parameters $D_{1}$ and $R_{1}$ are identified using the following expressions:

(10) $$D_{1}=\frac{\sigma_{b}m_{2}}{f_{1}\left({\dot{\varepsilon }}_{vp,02}\right)\left(m_{2}-m_{1}\right)}-\frac{\sigma_{s}m_{1}}{f_{1}\left({\dot{\varepsilon }}_{vp,2}\right)\left(m_{2}-m_{1}\right)}-R_{0}\;$$

(11) $$R_{1}=\frac{\sigma_{s}m_{2}}{f_{1}\left({\dot{\varepsilon }}_{vp,2}\right)\left(m_{2}-m_{1}\right)}-\frac{\sigma_{b}m_{1}}{f_{1}\left({\dot{\varepsilon }}_{vp,02}\right)\left(m_{2}-m_{1}\right)}+R_{0}\;$$

Equations (10) and (11) were obtained for γ=0 inserted to (9) and computations for the initial plastic strains ($\varepsilon_{vp}=0.2\%$) and the plastic strains for which the hardening is saturated ($\varepsilon_{vp}=2\%$), respectively. The value of ${\dot{\varepsilon }}_{vp,2}$ was computed numerically. ${\dot{\varepsilon }}_{vp,02}$ was assumed to be equal to ${\dot{\varepsilon }}_{02}$, as stated before.

### 2.5. DIC Enhancement of the Identification Procedure

**Figure 7:**
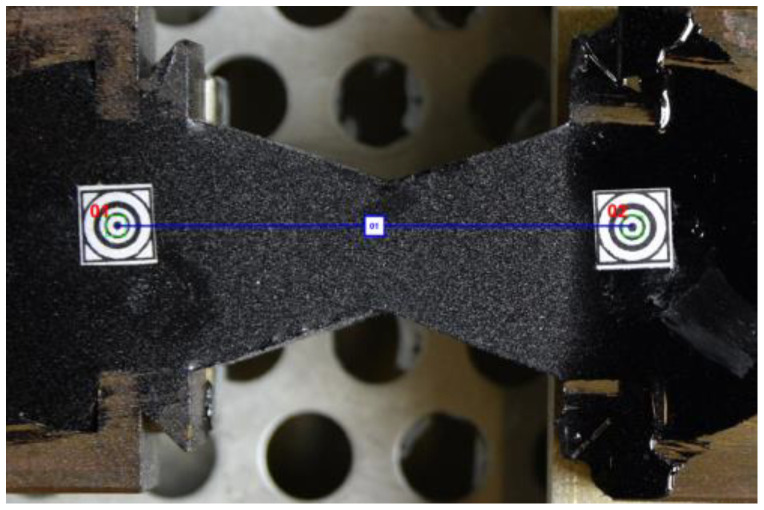
A single pair of markers used for the elongation measurement with the DIC technique.

The specimen shape is shown in Figure 2. Slightly different shapes are also used in ductilometer tests. All of them allow for the approximated uniaxial tensile tests. In fact, the elongation measurements refer to the points in the central axis of the specimen. In terms of the DIC technique, it is equivalent to a single pair of markers used as shown in Figure 7.

**Figure 8:**
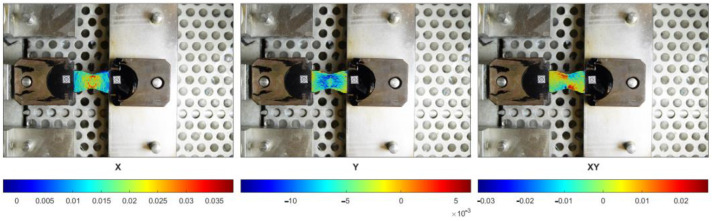
The maps of the strain tensor components: $\varepsilon_{xx}$, $\varepsilon_{yy}$, $\varepsilon_{xy}$, consecutively.

In reality, the strain state of the specimen is far from being homogeneous. In Figure 8, the maps of three components of the strain tensor ($\varepsilon_{xx}$*,* $\varepsilon_{yy}$*,* $\varepsilon_{xy}$) are presented. In the uniaxial tensile test, one is particularly interested in the $\varepsilon_{xx}$ component.

**Figure 9:**
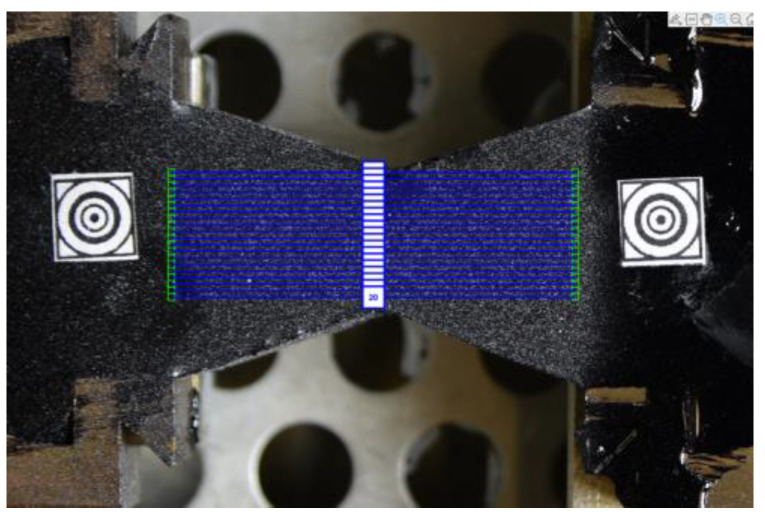
Twenty virtual tensometers placed along the specimen cross-section.

It can be easily noticed that even along the central axis and in its closest neighborhood, the $\varepsilon_{xx}$ component is not equal. In order to improve the accuracy of the measurements, we used the elongations of the specimen averaged along its cross-section as shown in Figure 9.

Technically, a number of virtual tensometers (we used 20 in this study) was applied. Obtained elongation values were averaged and used for the strain computations (see Figure 3).

**Figure 10:**
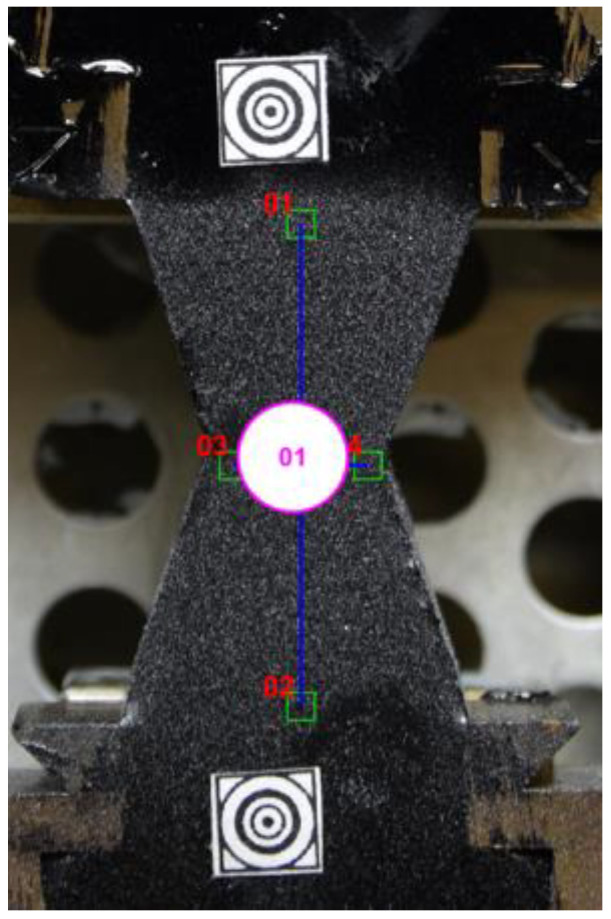
A pair of the perpendicular virtual tensometers used for the Poisson ratio assessment.

The DIC technique was also used for the Poisson ratio assessment, which could not be carried out using only the ductilometer test results. Namely, a pair of perpendicular virtual tensometers was placed on the specimen, as shown in Figure 10.

In this study, the value of approximately 0.35 was assessed, which can be also found in the literature [14]. We computed it averaging the values obtained for every three specimens and for every elongation rate.

Summing up, the enhancement of the standard methodology due to the DIC application consists of obtaining more reliable input data and its broader scope.

Specifically, we enhanced the methodology of Bodner–Partom model parameter identification with the DIC for several reasons:

- We were capable of computing the strain state not only between the two outermost points along the central axis of the specimen (as is the case of the standard ductilometer test results) but also within its whole surface and computing the averaged response along the specimen cross-section, consequently. In such a manner, we could eliminate a typical hidden assumption on the homogeneity of the specimen response along its cross-section;
- The application of the DIC technique allows for the recording of the laboratory test results with a user-defined frequency, which can overcome the limitations of the testing machine used.
- The parameters of the B–P model identified using the standard methodology would be enough for the further numerical tests only in 1D. Application of the DIC also enables the identification of the Poisson ratio, which is crucial to numerical modeling in 2D and 3D space.

## 3. Results

### 3.1. Results of the Identification Procedure

Below, we present the obtained values of Bodner–Partom parameters for the standard experiment type (Table 2) and its modified version due to DIC application (Table 3). Let us remind that the specimen in the first test was immersed in water at a temperature of 10 °C, whereas the second test was performed without the presence of water in a ductilometer, at a temperature equal to 19.5 °C.

One can observe an obvious dependence of the obtained results on the temperature at which the test was performed. Even the elastic response, which is characterized using Young’s modulus, is significantly different. A drop of about one order of magnitude (89.5%) is observed for this parameter at the room temperature. A similar relationship can be noticed for the parameters $D_{1}$ (a drop of 89.9%), $R_{0}$ (a drop of 89.5%), $R_{1}$ (a drop of 90.0%) that characterize the initial ($R_{0}$) and limiting ($D_{1}$ and $R_{1}$) values of the hardening functions. Higher values of parameters $m_{1}$ (increase of 89.7%) and $m_{2}$ (increase of 90.4%) denote earlier saturation of these functions at the higher temperature (19.5 °C). Only the parameter n does not exhibit a strong sensitivity to the temperature at the analyzed range (increase of 0.7%). As is discussed in the next section, the Bodner–Partom model is highly sensitive to the parameter n changes. Thus, one should not assume that this value is constant at every temperature, even in such a narrow temperature range (10÷19.5 °C).

Performing more standard experiments (with the presence of water), one could investigate the relationship between the model parameters and the temperature. We performed the ductilometer test at the temperature of 10 °C because this is a standard value used [25].

### 3.2. Numerical Results

**Figure 11:**
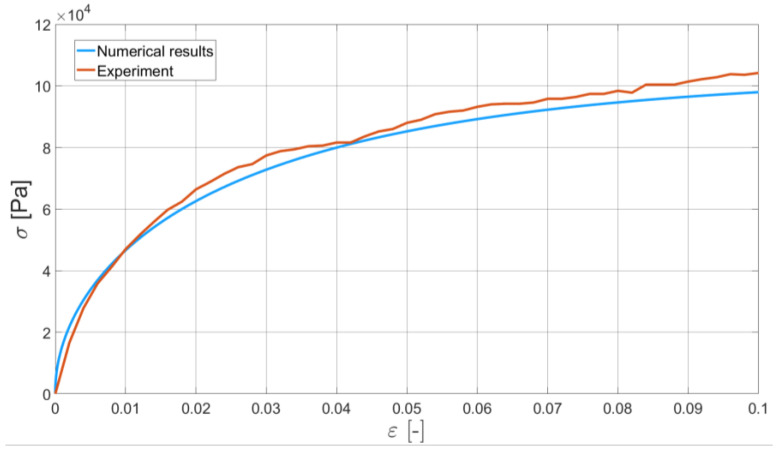
The comparison of the real experiment and its numerical simulation. Elongation rate is equal to 6 mm/min and the DIC-enhanced results are presented for the experiment.

**Figure 12:**
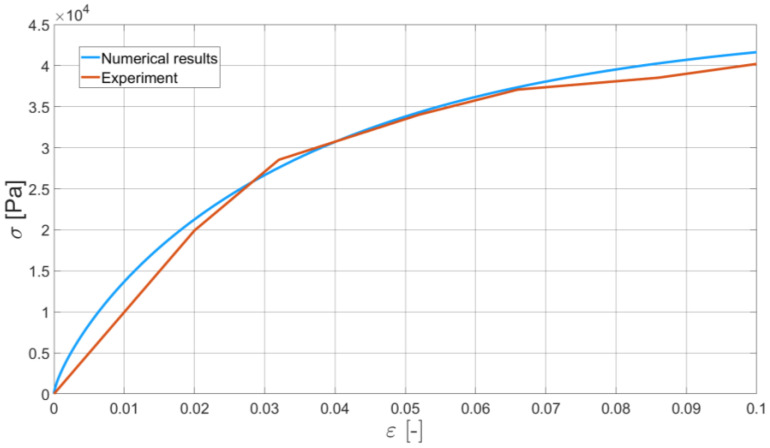
The comparison of the real experiment and its numerical simulation. Elongation rate is equal to 50 mm/min and the DIC-enhanced results are presented for the experiment.

In order to validate the Bodner–Partom model with application to bitumen binder, we present the comparison of two of the experiments and their numerical counterparts. Namely, we identified Bodner–Partom model parameters and used them for the self-developed finite element procedure. As an exemplary result of such a comparison, we present the DIC-enhanced ductilometer tests performed with the elongation rates equal to 6 mm/min and 50 mm/min (see Figure 11 and Figure 12, respectively).

For these specific tests, the differences between the experimental and numerical results are of the maximum order of 10%. A similar characteristic can be also observed for the other elongation rates. Some discrepancies between the measured and modeled material response can be explained by a constant ductilometer measurement frequency. For the higher elongation rates, at the intervals with the larger gradients of the σ−ε curve observed, the measurement frequency may be too small to capture the nonlinear material response to the applied monotonic strain.

The obtained numerical results, in some sense, smoothen the bitumen binder response. No oscillations typical of real experiments were observed. The initial results confirm the applicability of the Bodner–Partom model to the numerical analysis of bitumen binder.

The sensitivity analysis of the model revealed that the final results are particularly sensitive to the value of parameter n. Thus, special attention needs to be paid to its identification. The improvement can be obtained in a twofold manner. First of all, a number of experiments with various elongation rates can be substantially increased. Secondly, algorithms other than Marquardt–Levensberg [28] can be applied to increase the reliability of the function (see Figure 5) approximation.

Additionally, the manufacturer of the analyzed neat bitumen 35/50 provides some functional properties in [29]. For instance, the results of the dynamic shear rheometer tests (DSR) performed on the specimens subjected in advance to the rolling thin film oven test (RTFOT) are presented in [29].

Complex stiffness modulus |G ∗| describes the ability of the bitumen binder to resist the applied load. It can be further decomposed into the elastic and viscous part that describe the mode of material response, respectively. The phase angle δ defines a dominant component. When it is equal to 0°, the material behaves elastically. Approaching 90°, it denotes that the material behaves like a Newtonian fluid. Both mechanical parameters are functions of temperature and load rate. In ([29], p. 86), the results for two frequencies are presented. It can be observed that, in particular, the complex stiffness modulus increases with the increase in load rate.

Presented (see Section 3.2 in [29]) results define the range of the applicability of the analyzed bitumen binder type to some extent. As suggested by the manufacturer [29], it can be used in the case of the base and binder courses made of asphalt concrete. Its application to wearing courses is not recommended.

## 4. Discussion

In this study, the initial results of the Bodner–Partom model’s application to the numerical modeling of bitumen binder are presented. The whole process is enhanced with a DIC technique, which provides a more reliable input data for the model parameters identification. The obtained results confirm that the adopted constitutive model can be applied to the modeling of neat bitumen binder in the analyzed temperature range. As demonstrated in Figure 10 and Figure 11, the Bodner–Partom model can properly describe the behavior of such a complex material as binder bitumen with good agreement with the experimental results. The difference between the modeled and measured response of bitumen binder to the applied elongation rates (see Figure 10 and Figure 11) is of a maximum order of 10%. This confirms the applicability of the proposed model. The numerical efficiency of the time-stepping algorithm should additionally be underlined. The explicit Euler integration scheme is free of Newton–Raphson iterations, which makes the finite element code faster.

Looking at the identified Bodner–Partom parameters, which were assessed at different temperatures due to DIC limitations (water in ductilometer), one can observe a strong relationship between their values and the temperature. It makes identified parameter application limited to a specific temperature. In order to overcome this problem, so-called shift factors are used [5] to transfer the solution obtained at a given temperature to another. Alternatively, one can use the extensions of the Bodner–Partom model that accounts for thermal effects.

Carrying out a greater number of standard ductilometer experiments (with the presence of water) at various temperatures, one could study the relationship between the model parameters and the temperature.

As mentioned at the end of Section 3.2, the model is very sensitive to the value of parameter n. This part of the identification procedure also needs to be processed in order to increase the accuracy of the whole procedure.

We address this study to the further multiscale analyses of the selected asphalt mixtures. With the Bodner–Partom parameters identified, it is possible to account for this model for the binder phase of the asphalt mixture and apply other (e.g., linear elastic) models for the aggregate and possible additives. Finally, the effective macroscale response of the whole asphalt mixture can be studied. A number of possible multiscale approaches [5,14] can be applied.

## 5. Conclusions

Concluding the findings of this study:

- The constitutive Bodner–Partom model is feasible of capturing the response of the bitumen binder properly in the range of small strains (this restriction is justified with a future application of the obtained results to the mechanistic pavement design method);
- The physically based identification procedure of model parameters [22,23] can be also successfully applied to bitumen binder;
- In the specific case of neat bitumen binder 35/50, the differences between the experimental and numerical results are at the acceptable level (maximum error is of order of 10%);
- The DIC technique demonstrates its usefulness in the enhancement of the identification procedure due to the increase of the input data quality;
- Further research efforts should be made in order to increase the accuracy of the crucial model parameter’s identification and the application of the extended versions of the Bodner–Partom model that account for the thermal effects;
- The obtained results can be applied to the multiscale analyses of different asphalt mixtures.

## Figures and Tables

**Table 1:** Neat bitumen binder 35/50 parameters.

Penetration at 25 °C (0.1 mm)	Softening Point R&B (°C)	Fraas Breaking Point (°C)	Ductility (cm)	Specific Gravity (kg/m^3^)
45	55.8	−16	>100	1023

**Table 2:** Bodner–Partom model parameters of bitumen binder 35/50 (at a temperature of 10 °C).

*E* (Pa)	*ν* (-)	*n* (-)	*D*_0_ (1/s)	*D*_1_ (Pa)	*R*_0_ (Pa)	*R*_1_ (Pa)	*m*_1_ (1/Pa)	*m*_2_ (1/Pa)
1 × 10^7^	0.35	0.1611	1	2.2541 × 10^4^	1.2784 × 10^5^	6.1822 × 10^5^	3.2 × 10^−4^	0.0803

**Table 3:** Bodner–Partom model parameters of bitumen binder 35/50 (at the temperature of 19.5 °C).

*E* (Pa)	*ν* (-)	*n* (-)	*D*_0_ (1/s)	*D*_1_ (Pa)	*R*_0_ (Pa)	*R*_1_ (Pa)	*m*_1_ (1/Pa)	*m*_2_ (1/Pa)
1.05 × 10^6^	0.35	0.1623	1	2.2702 × 10^3^	1.3423 × 10^4^	6.1706 × 10^4^	0.0031	0.8330

## Data Availability

Not applicable.
